# QDPR rs3733570 polymorphism is associated with type 2 diabetes and diabetic kidney disease accompanied by hyperlipidemia in the Chinese Han population

**DOI:** 10.3389/fmed.2026.1811028

**Published:** 2026-05-12

**Authors:** Minda Dong, Yeqiang Liu, Zhijie Pu, Hong Shen, Zhiguo Li

**Affiliations:** 1 The Hebei Key Lab for Organ Fibrosis, School of Public Health, International Science and Technology Cooperation Base of Geriatric Medicine, North China University of Science and Technology, Tangshan, China; 2 Department of Endocrinology, Tangshan Central Hospital, Tangshan, China; 3 Department of Endocrinology, Yutian County Hospital, Tangshan, China; 4 Modern Technology and Educational Center, North China University of Science and Technology, Tangshan, China

**Keywords:** diabetic kidney disease, *QDPR*, single nucleotide polymorphism, stratified analyses, type 2 diabetes mellitus

## Abstract

**Background:**

Genetic factors play important roles in the development of type 2 diabetes mellitus and its complications. Diabetic kidney disease, a common microangiopathic complication of diabetes, is linked to an increased risk of major cardiovascular events and overall mortality. Tetrahydrobiopterin deficiency is implicated in DKD pathogenesis, and the *QDPR* gene is critical for maintaining BH4 homeostasis. The present study was designed to screen single nucleotide polymorphisms in the *QDPR* coding region, and assess their associations with T2DM and DKD.

**Methods:**

Genetic analysis for SNPs in the coding region was conducted using a systematic screening approach. Direct sequencing and statistical analysis tools were employed to identify potentially functional SNPs. Following this, rs3733570 was selected based on its potential biological relevance and frequency in the target population. A total of 1,844 participants from Kai Luan General Hospital (Tangshan, China) were included for validation. Genotyping of *QDPR* rs3733570 was performed using polymerase chain reaction-restriction fragment length polymorphism (PCR-RFLP) in 690 healthy controls, 818 patients with T2DM, and 336 patients with DKD.

**Results:**

Regression analysis showed that the rs3733570 AA genotype was associated with increased risk of T2DM (OR = 1.33, 95% CI: 0.99–1.79, *p* < 0.05). In stratified analysis, carriers of the AA genotype with dyslipidemia had a higher risk of DKD (OR = 1.80, 95% CI: 1.03–3.16, *p* < 0.05).

**Conclusion:**

The *QDPR* rs3733570 AA genotype may increase susceptibility to T2DM and, in the presence of dyslipidemia, to DKD in the Chinese Han population. After adjustment, the AA genotype showed a borderline association with T2DM risk (OR = 1.33, 95% CI: 0.99–1.79, *p* = 0.057).

## 1 Introduction

The global prevalence of diabetes mellitus (DM) represents a major public health challenge, imposing an increasing socioeconomic burden worldwide (1, 2). More than 90% of DM cases are attributed to type 2 diabetes mellitus (T2DM), a complex metabolic disorder characterized by high genetic susceptibility and associated with substantial risks to human health and longevity. Epidemiological studies have shown that a family history of T2DM is associated with an approximately 50% increased risk of incident T2DM compared with no family history (3). Among the complications of diabetes, diabetic kidney disease (DKD) is particularly clinically significant, being one of the most common microvascular complications and a leading cause of end-stage renal disease (ESRD). Despite advances in clinical management, therapeutic options for DKD remain limited, underscoring the urgent need to identify novel targets for the prevention and treatment of T2DM and its renal complications (4).

Tetrahydrobiopterin (BH4) is an essential cofactor for nitric oxide synthase (NOS), playing a critical role in maintaining endothelial function and vascular homeostasis. Overexpression of endothelial NOS (eNOS) in the absence of adequate intracellular BH4 leads to eNOS uncoupling and increased superoxide anion production (5). Additionally, dihydrobiopterin (BH2) can competitively bind to NOS, further promoting uncoupling and excessive generation of reactive oxygen species (ROS) (6, 7). Evidence suggests that an imbalance between BH4 and eNOS contributes significantly to oxidative stress in DKD pathogenesis (8, 9). ROS overproduction can impair insulin signaling, promoting insulin resistance, and inducing pancreatic β-cell dysfunction and apoptosis (10). Beyond its role in NOS function, BH4 serves as a cofactor for aromatic amino acid hydroxylases, including phenylalanine hydroxylase (PAH), tyrosine hydroxylase (TH), and tryptophan hydroxylase (TPH) (11). These enzymes are essential for the biosynthesis of neurotransmitters such as serotonin and dopamine, which have been implicated in renal physiology and may influence DKD onset and progression.

The *QDPR* gene encodes quinoid dihydropteridine reductase, a key enzyme involved in the maintenance of BH4 homeostasis. It catalyzes the reduction of quinoid dihydrobiopterin (qBH2) back to BH4 and represents the only rate-limiting step in the BH4 recycling pathway (12). Our previous work demonstrated that *QDPR* overexpression downregulates *transforming growth factor-β1* (*TGF-β1*) expression in human kidney 293 T cells (13) and attenuates oxidative stress while promoting autophagy (13, 14). Given the well-established roles of *TGF-β1* signaling, oxidative stress, and autophagy in DKD progression, these findings indicate that *QDPR* may serve as a pivotal regulator in DKD pathophysiology.

Genetic factors also critically influence susceptibility to T2DM and DKD. Single nucleotide polymorphisms (SNPs), the most common form of genetic variation, are stable and widely distributed across the genome, making them valuable markers for investigating the genetic basis of complex diseases (15, 16). Among the identified susceptibility genes, SLC11A1 variants rs3731865 and rs17235416 show strong associations with T2DM across multiple populations (17, 18). These risk alleles may impair pancreatic β-cell function by mediating cellular iron overload, inducing oxidative stress and inflammation, and exacerbating insulin resistance. In addition, RAC1 rs7784465 has been reported to be closely associated with impaired redox homeostasis, increased risk of T2DM, and hyperglycemia, with the T allele generally conferring protective effects (19). Similarly, genetic variations in *QDPR* could influence BH4 homeostasis and impact DKD risk, emphasizing its potential as a therapeutic target. Disruption of *QDPR* function not only impairs BH4 stability but is also implicated in inherited metabolic disorders such as phenylketonuria, highlighting its critical role in systemic metabolic regulation.

Collectively, these findings underscore the central role of BH4 metabolism and *QDPR* function in the pathogenesis of T2DM and DKD, suggesting that genetic and molecular modulation of this pathway may offer promising avenues for therapeutic intervention.

## 2 Materials and methods

### 2.1 Sample collection

A hospital-based case–control study was conducted at Kai Luan General Hospital (Tangshan, China) from March 2012 to July 2017. A total of 1,844 unrelated adult participants of Han Chinese ethnicity and long-term residents of Hebei Province were enrolled. The study population included 818 patients with T2DM without DKD, 336 patients with DKD, (both groups recruited from the Department of Endocrinology and Metabolism), alongside 690 healthy controls recruited from the Physical Examination Center. In accordance with *Standards of Care in Diabetes* criteria (20) and previous studies, T2DM was diagnosed if participants met at least one of the following conditions: (a) random plasma glucose ≥11.1 mmol/L with classic hyperglycemic symptoms; (b) fasting plasma glucose ≥7.0 mmol/L; (c) 2-h plasma glucose ≥11.1 mmol/L during an oral glucose tolerance test; or (d) currently normal glucose levels with ongoing antidiabetic treatment. Patients were eligible for inclusion in the T2DM group if they had a physician-confirmed diagnosis of T2DM and provided written informed consent. The exclusion criteria for the T2DM group were as follows: (a) advanced renal dysfunction, defined as an estimated glomerular filtration rate (eGFR) < 30 mL/min/1.73 m^2^, or a history of dialysis or renal transplantation; and (b) advanced or decompensated diabetes, diabetic coma, immune-mediated or idiopathic type 1 diabetes, gestational diabetes, maturity-onset diabetes of the young (MODY), diseases affecting the exocrine pancreas (e.g., pancreatitis, pancreatic trauma, pancreatectomy, or pancreatic tumors), hereditary pancreatic disorders, or other endocrine disorders. The diagnosis of DKD was established in accordance with the Clinical Guidelines for the Prevention and Treatment of Diabetic Kidney Disease in China, together with consensus statements from the American Diabetes Association and the National Kidney Foundation. Patients were included if they had a definitive history of T2DM combined with either: (a) persistent renal abnormalities for over 3 months (21), defined as a urinary albumin-to-creatinine ratio (UACR) ≥ 30 mg/g (or 3 mg/mmol) and an estimated glomerular filtration rate (eGFR) < 60 mL/min/1.73 m^2^ (22); or (b) renal biopsy results consistent with DKD pathology. For atypical cases, a renal biopsy was mandatory to confirm the diagnosis and exclude non-diabetic kidney disease (NDKD). The study was approved by the Ethics Committee of Kai Luan General Hospital (Approval No. KLGH-2006-05) and registered in the Chinese Clinical Trial Registry (ChiCTR-TNRC-11001489).

### 2.2 Sample size estimation

**Figure 1:**
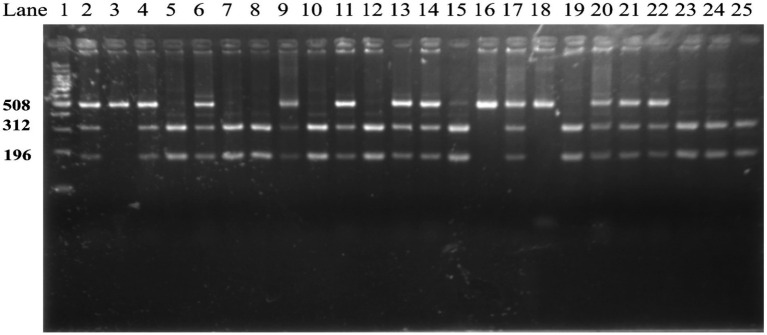
Genotyping of samples by RFLP analysis. Lane 1 (M): 100 bp DNA ladder, GA genotype: lanes 2, 4, 6, 9, 11, 13, 14, 17, 20–22; AA genotype: lanes 3, 16, 18; GG genotype: lanes 5, 7, 8, 10, 12, 15, 19, 23–25.

**Figure 2:**
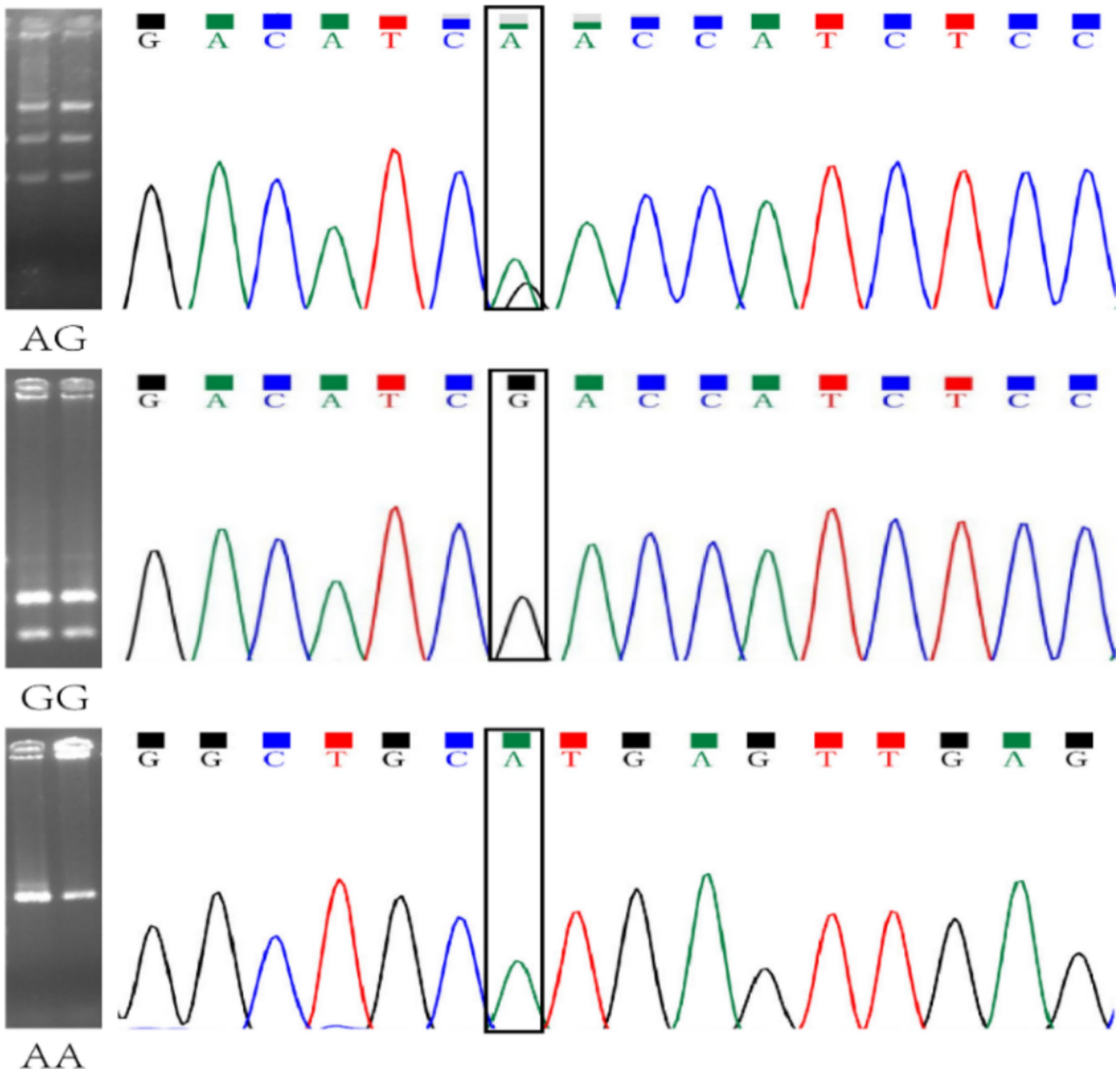
Confirmation of the RFLP results by sequencing.

Sample size estimation was performed using G*Power version 3.1 for a chi-square test of contingency tables (df = 2). Assuming a small effect size (Cohen’s *w* = 0.10), a significance level of 0.05, and a statistical power of 80%, the minimum required total sample size was estimated to be 964. The actual sample size in the present study exceeded this requirement (Figures 1, 2).

### 2.3 Biochemical parameters evaluation

All participants underwent a comprehensive medical history review and physical examination. Demographic data including age, sex, duration of diabetes were collected from medical records from the previous year. Additional clinical data included serum creatinine, uric acid, hemoglobin, red blood cell count, white blood cell count, and other biochemical indicators were tested at the central laboratory of Kai Luan General Hospital using standardized clinical testing protocols. Dyslipidemia was defined according to established criteria (23): Hypercholesterolemia (TC ≥ 5.2 mmol/L), triglycerides (TG < 1.7 mmol/L); Hypercholesterolemia (TC ≥ 5.2 mmol/L and TG < 1.7 mmol/L); Mixed hyperlipidemia (TC ≥ 5.2 mmol/L, TG ≥ 1.7 mmol/L); Hypo-HDL cholesterolemia (HDL-C < 1.0 mmol/L). Systolic and diastolic blood pressure (SBP and DBP) were measured using a standardized method. Three readings were obtained with participants in a seated position using a conventional sphygmomanometer. Hypertension was defined as a systolic blood pressure ≥140 mmHg and/or a diastolic blood pressure ≥90 mmHg.

### 2.4 Direct sequencing of the QDPR coding region

To identify potential SNPs in the *QDPR* gene, we first performed a pilot screening study in a subset of participants, including 25 individuals from the NC group and 25 individuals from the DKD group. The coding region of *QDPR* was analyzed by direct sequencing. Briefly, six target regions were amplified by polymerase chain reaction (PCR) using the primer sets listed in Table 1, and the PCR products were subjected to Sanger sequencing by GENEWIZ.

**Table 1:** Searching for SNPs associated with DKD by direct sequencing.

SNP	TYPE	NC (Maj/Het/Min)	DKD (Maj/Het/Min)	Position	Primer sequence	PCR product
Exon 2						
rs1031327	G-A	18/4/3	11/9/5	Chr4:17486621 (GRCh38.p14)	F CTCTTACCTGTCTCCACTC	578 bp
rs2518607	G-A	21/3/1	10/15/0	Chr4:17512513 (GRCh38.p14)	R GGAAGAACATACAGCCAGT	
rs10604	A-G	2/12/11	6/9/10	Chr4:17486509 (GRCh38.p14)		
rs1049581	A-G	10/14/1	15/10/0	Chr4:17486911 (GRCh38.p14)		
rs1049601	G-A	13/11/1	16/9/0	Chr4:17486723 (GRCh38.p14)		
Exon 3						
rs2305316	G-A	20/6/0	8/20/1	Chr4:17512135 (GRCh38.p14)	F GATGCTCATGTCTCTACCTT	650 bp
rs2597775	C-T	12/7/6	10/16/3	Chr4:17501759 (GRCh38.p14)	R ATCCTCCAATGCCTGAATAG	
rs104893867	C-T	12/17/1	16/13/0	Chr4:17504404 (GRCh38.p14)		
rs764033550	G-T	12/14/0	14/14/1	Chr4:17490754 (GRCh38.p14)		
rs891408281	G-T	10/16/0	10/18/1	Chr4:17490688 (GRCh38.p14)		
Exon 4						
rs940829956	A-G	10/10/5	14/15/1	Chr4:17512037 (GRCh38.p14)	F TTGCCAGTGTATAGGTAAGG	642 bp
rs3733570	G-A	4/5/16	1/2/27	Chr4:17501810 (GRCh38.p14)	R ATTCATTCCAGTGTAGA	
rs2315248	G-A	9/14/2	10/19/1	Chr4:17504541 (GRCh38.p14)		
Exon 5						
rs2305313	T-C	18/7/2	16/28	Chr4:17492550 (GRCh38.p14)	F TGTCATGTCTCCTCCACTT	698 bp
rs2597772*	C-T	0/0/17	0/0/28	Chr4:17492431 (GRCh38.p14)	R TCCAGAACTGTGAGCAATAC	
Exon 6						
rs1309099253	G-A	2/8/16	1/2/25	Chr4:17501858 (GRCh38.p14)	F AATGTGGCTCCTTCTGTT	636 bp
					R CTCAGCAGGTCAATAATGTG	

*Homozygous mutations in normal humans.

### 2.5 Polymerase chain reaction-restriction fragment length polymorphism (PCR-RFLP)

The selected variant was genotyped by polymerase chain reaction-restriction fragment length polymorphism (PCR-RFLP). The flanking sequence surrounding rs3733570 was obtained from the NCBI dbSNP database. Primers were designed using Primer Premier 6.0 and synthesized by Suzhou GENEWIZ Biotechnology Co., Ltd. (Suzhou, China). The details of the primer sequence were as follows: forward: 5′-AAGGCATACTAGTACAAGTGGTAA-3′ and reverse: 5′-TGTGTTCCCTCAATGTTTCAGT-3′. Peripheral blood samples were collected in EDTA-anticoagulated tubes, and genomic DNA was extracted from whole blood using a standard protocol. PCR amplification was performed in a final reaction volume of 25 μL, containing 2.0 μL genomic DNA (50 ng), 1.0 μL of each primer (10 μM), 10.0 μL of 2 × Taq PCR Master Mix (Thermo Fisher Scientific, United States), and 11.0 μL nuclease-free water. Amplification was performed on an Applied Biosystems GeneAmp PCR System 9700 under the following conditions: initial denaturation at 94 °C for 5 min; 30 cycles of denaturation at 94 °C for 30 s, annealing at 52 °C for 30 s, and extension at 72 °C for 30 s; followed by a final extension at 72 °C for 10 min. PCR products were further digested with TaqI restriction enzyme (New England Biolabs, United States) at 65 °C for 3 h. The digested products were separated by electrophoresis on a 2% agarose gel stained with ethidium bromide and visualized under ultraviolet illumination using a 100-bp DNA ladder as the size marker. After digestion, the A allele yielded a 508-bp fragment, whereas the G allele generated 312-bp and 196-bp fragments. Accordingly, the expected banding patterns were 508 bp for the AA genotype, 508, 312, and 196 bp for the GA genotype, and 312 and 196 bp for the GG genotype. Genotype and allele frequencies were calculated by direct counting. Deviation from Hardy–Weinberg equilibrium (HWE) in the control group was assessed using the chi-square test.

### 2.6 Statistical analysis

All statistical analyses were conducted using IBM SPSS Statistics version 27.0 (IBM Corp., Armonk, NY, USA). Normally distributed continuous variables are presented as mean ± standard deviation (SD), whereas non-normally distributed variables, such as serum creatinine and uric acid, are expressed as median with interquartile range (IQR). Comparisons between two groups of normally distributed continuous variables were performed using the Student’s t-test, whereas comparisons among more than two groups were conducted using one-way analysis of variance (ANOVA) followed by Bonferroni *post hoc* tests for pairwise comparisons. Non-normally distributed variables were compared using the Mann–Whitney U test or Kruskal–Wallis test, as appropriate. Associations between *QDPR* polymorphisms and the risks of T2DM and DKD were evaluated using logistic regression analysis, and the results are presented as odds ratios (ORs) with 95% confidence intervals (CIs). A two-tailed *p* < 0.05 was considered statistically significant. Sample size estimation and power analysis were performed using G*Power software.

## 3 Results

### 3.1 Identification of candidate SNPs in the QDPR coding region

During variant screening, all six exons, the 5′ and 3′ untranslated regions (UTRs), and the 5′ flanking region of the *QDPR* gene, spanning approximately 20 kb of genomic DNA, were examined. To explore the potential association between *QDPR* variants and DKD, the coding region was initially screened in 25 participants from the healthy control group and 25 participants from the DKD group. As shown in Table 1, a total of 16 SNPs were identified across the two groups. Notably, exon 1 could not be successfully amplified, possibly because of its high GC content. Among the detected variants, rs3733570 emerged as the most promising candidate and was therefore selected for subsequent analysis.

### 3.2 Clinical characteristics of the study population

The clinical characteristics of the study participants are summarized in Table 2. The study population included 690 healthy controls, 818 patients with T2DM without DKD, and 336 patients with DKD. The proportions of male participants were 65.4% in the control group, 56.7% in the T2DM group, and 70.5% in the DKD group. No marked difference was noticed between the T2DM (62.36 ± 9.82 years) and DKD (61.66 ± 9.22 years) group concerning age. However, the age at onset of T2DM differed significantly between the two groups (46.78 ± 10.71 vs. 53.59 ± 10.71 years, *p* < 0.001). At the same time, diabetes duration and metabolic parameters, including fasting plasma glucose (FPG), triglycerides (TG), total cholesterol (TC), high-density lipoprotein cholesterol (HDL-C), and low-density lipoprotein cholesterol (LDL-C), also differed significantly among the groups (*p* < 0.001). The prevalence of dyslipidemia was 28% in the NC group, 40% in the T2DM group, and 54% in the DKD group. Compared with the T2DM group, the DKD group showed a more pronounced dyslipidemic profile, characterized by higher TG and VLDL-C levels and lower HDL-C levels (*p* < 0.05). SBP and DBP were significantly higher in the DKD group than in the T2DM and NC groups. Number of patients with hypertension was significantly higher in the DKD group than in control and in the T2DM groups. As expected, renal function was significantly worse in patients with DKD, as reflected by higher serum creatinine and UACR levels and lower eGFR values than those in the NC and T2DM groups (all *p* < 0.001). Moreover, compared with the control group, patients with DKD had lower hemoglobin levels and red blood cell counts, indicating anemia-related changes, as well as higher white blood cell counts and C-reactive protein levels, suggesting an enhanced inflammatory state. Among patients with DKD, 64% had peripheral vascular disease and 63% had diabetic peripheral neuropathy. Other diabetic complications included diabetic retinopathy (44.8%), diabetic foot syndrome (6.3%), and coronary artery disease (37.3%). Patients with these complications had a significantly longer duration of T2DM (*p* < 0.05).

**Table 2:** Demographics and clinical characteristics of the studied population.

Variable	NC	T2DM	DKD	*p*	*p^a^*	*p^b^*	*p^c^*
Number	690	818	336				
Age (years)	70.10 ± 7.90	62.36 ± 9.82	61.66 ± 9.22	<0.001	<0.001	<0.001	0.233
Male [*n* (%)]	478 (70.1)	448 (54.6)	209 (62.2)	<0.001	<0.001	<0.001	0.029
Duration (year)	0	9.05 ± 6.54	15.02 ± 7.05	<0.001	<0.001	<0.001	<0.001
Age of Onset (year)	–	53.59 ± 10.71	46.78 ± 10.07	<0.001			
FPG (mmol/L)	4.95 ± 0.91	10.64 ± 5.28	11.35 ± 6.10	<0.001	<0.001	<0.01	0.015
BUN (mmol/ L)	6.05 ± 2.36	6.06 ± 2.73	9.49 ± 6.71	<0.001	0.974	<0.001	<0.001
UA (μmol/L)	311.50 (252.371)	288 (292.25433)	366.5 (237.355)	<0.001	0.022	<0.001	<0.001
SCR (μmol/L)	60 (49.71)	53 (44.65)	81 (57.150)	<0.001	<0.001	<0.001	0.761
TC (mg/dL)	4.60 ± 1.13	4.94 ± 1.37	5.49 ± 1.89	<0.001	<0.001	<0.001	0.280
TG (mg/dL)	1.28 ± 0.96	1.99 ± 1.74	2.41 ± 2.54	<0.001	<0.001	<0.001	0.394
HDL-C (mg/dL)	1.09 ± 0.30	1.07 ± 0.30	1.09 ± 0.30	0.290			
LDL-C (mg/dL)	2.27 ± 0.83	2.43 ± 0.85	2.54 ± 1.02	<0.001	<0.001	<0.001	0.073
VLDL-C (mg/dL)	0.27 ± 0.31	0.42 ± 0.43	0.49 ± 0.54	<0.001	<0.001	<0.001	0.005
APOA (g/L)	1.01 ± 0.21	1.04 ± 0.19	1.06 ± 0.23	0.001	0.005	0.001	0.255
APOB (g/L)	0.80 ± 0.17	0.88 ± 0.20	0.96 ± 0.27	<0.001	<0.001	<0.001	<0.001
APOA/APOB	1.31 ± 0.33	1.26 ± 0.35	1.17 ± 0.42	<0.001	0.004	<0.001	<0.001
SBP (mmHg)	136.16 ± 20.12	136.24 ± 20.10	146.99 ± 23.90	<0.001	0.967	<0.001	<0.001
DBP (mmHg)	80.47 ± 12.23	80.88 ± 10.71	84.18 ± 12.20	<0.001	0.602	<0.001	<0.001
WBC (10^9/L)	7.20 ± 2.86	7.53 ± 2.72	7.98 ± 2.84	<0.001	0.026	<0.001	0.012
RBC (10^12/L)	4.33 ± 0.61	4.55 ± 0.62	4.21 ± 0.79	<0.001	<0.001	<0.001	<0.001
Hb (g/L)	135.90 ± 18.91	139.39 ± 20.18	127.54 ± 25.18	<0.001	0.002	<0.001	<0.001
Hyperlipidemia [*n* (%)]	107 (28)	330 (40)	181 (54)	<0.001	<0.001	<0.001	<0.001
Hypertension [*n* (%)]	230 (60)	511 (62.2)	277 (83)	<0.001	0.745	<0.001	<0.001
Cerebrovascular diseases [*n* (%)]	112 (29)	300 (36.5)	172 (58.1)	<0.001	0.023	<0.001	<0.001
Coronary heart disease [*n* (%)]	130 (34)	302 (36.7)	124 (37.3)	0.746			
Diabetic peripheral neuropathy [*n* (%)]	–	250 (30.4)	210 (63.2)	<0.001			
Diabetic peripheral vascular disease [*n* (%)]	–	304 (37)	213 (64)	<0.001			
Diabetic retinopathy [*n* (%)]	–	106 (12.9)	149 (44.8)	<0.001			
Diabetic foot [*n* (%)]	–	9 (1.09)	21 (6.32)	<0.001			

*p*^a^: T2DM VS NC; *p*^b^: DKD VS NC; *p*^c^: DKD VS T2DM.

### 3.3 Genotype and allele distributions of QDPR rs3733570

Detailed genotypic and allelic distributions of rs3733570 in the *QDPR* gene are shown in Table 3. Chi-square test (*χ^2^*) was performed to assess whether the genotype distribution of the *QDPR* rs3733570 locus in the healthy controls group conformed to HWE. Statistical analysis results showed no statistically significant difference (*p* > 0.05), indicating that the healthy controls cohort in the present study was representative of the general population with no significant selection bias, and thus suitable for subsequent genetic association analyses. A significant association was observed between the *QDPR* rs3733570 G/A polymorphism and susceptibility to T2DM. Under the codominant model, individuals with the AA genotype had a significantly higher risk of T2DM than those with the GG genotype (OR = 1.37, 95% CI: 1.03–1.82, *p* < 0.05). The comparison between the GG and GA genotypes showed a borderline trend (OR = 1.18, 95% CI: 0.93–1.50, *p* = 0.051). Under the dominant model, carriers of the GA + AA genotypes showed an increased risk of T2DM compared with GG carriers. In contrast, no statistically significant differences in genotype or allele frequencies were observed between the T2DM and DKD groups (*p* > 0.05), suggesting that rs3733570 may be associated with susceptibility to T2DM, but not with progression from T2DM to DKD in the present study.

**Table 3:** Genotype and allele distribution of QDPR gene rs3733570 polymorphisms among the study groups.

Genetic models	NC [*n* (%)]	T2DM [*n* (%)]	DKD [*n* (%)]	*p^a^*	OR (95%CI)	*p^b^*	OR (95%CI)	*p^c^*	OR (95%CI)
	690	818	336						
Codominant model									
GG	219 (31.7)	215 (26.2)	89 (26.4)						
GA	317 (45.9)	395 (48.2)	169 (50.4)	0.051	1.26 (0.99–1.61)	0.085	1.03 (0.96–1.43)	0.832	1.03 (0.96–1.40)
AA	154 (22.3)	208 (25.4)	78 (23.2)	0.026	1.37 (1.03–1.82)	0.240	1.16 (0.90–1.49)	0.589	0.90 (0.63–1.29)
Recessive model									
GA + GG	536 (77.6)	610 (74.4)	259 (77.2)						
AA	154 (22.3)	208 (25.4)	76 (22.6)	0.159	1.18 (0.93–1.50)	0.748	1.05 (0.77–1.43)	0.429	0.87 (0.65–1.15)
Dominant model									
GG	219 (31.7)	215 (26.2)	90 (26.8)						
AA+GA	471 (68.2)	603 (73.6)	245 (73)	0.020	1.23 (0.81–1.98)	0.085	0.77 (0.58–1.03)	0.943	1.01 (0.75–1.34)
Allele frequency									
G	378 (0.548)	414 (0.505)	167 (0.495)						
A	312 (0.452)	404 (0.495)	169 (0.505)	0.106	0.84 (0.69–1.03)	0.126	0.91 (0.79–1.04)	0.535	0.96 (0.84–1.09)

Data presented as numbers of patients (percentages). *p*^a^: T2DM VS NC; *p*^b^: DKD VS NC; *p*^c^: DKD VS T2DM.

### 3.4 Association of rs3733570 with T2DM in multivariable logistic regression analysis

Multiple logistic regression analysis was performed to identify independent risk factors for T2DM and DKD. Before multivariable analysis, univariate analyses were conducted to preliminarily identify variables showing significant differences (Supplementary Tables 1, 2).

The multiple logistic regression showed significant differences in TC, HDL-C, and WBC levels between carriers of the AA and GA genotypes (*p* < 0.05) (Table 4). After adjustment, individuals with the GA genotype exhibited a 1.33-fold increased risk of developing T2DM. In addition, abnormal LDL-C levels were independently associated with a significantly increased risk of T2DM (OR = 1.64, 95% CI: 1.33–2.03). As the regression analysis for diabetic kidney disease did not show statistical significance, the corresponding results are provided in Supplementary Table 3.

**Table 4:** Unconditional multifactorial logistic regression analysis of risk factors for type 2 diabetes mellitus.

Variables	*β*	S.E	Wald *χ*^2^	*p*	OR (95%CI′)
Codominant model					
Intercept	−1.833	0.337	0.698	0.404	
GG	0^b^				
GA	0.284	0.129	4.834	0.028	1.32 (1.03–1.71)
AA	0.289	0.151	3.634	0.057	1.33 (0.99–1.79)
TC	−0.297	0.088	11.355	0.001	0.74 (0.62–0.88)
TG	0.351	0.296	1.404	0.236	1.42 (0.79–2.53)
HDL-C	0.560	0.220	6.452	0.011	1.75 (1.13–2.69)
LDL-C	0.499	0.108	21.258	<0.001	1.64 (1.33–2.03)
VLDL-C	2.040	1.481	1.897	0.168	7.68 (0.42–14.05)
WBC	0.048	0.020	5.728	0.017	1.04 (1.00–1.09)
Dominant model					
Intercept	−1.833	0.337	0.698	0.404	
GG	0^b^				
AA+GA	−0.264	0.119	4.893	0.027	1.41 (1.03–1.71)
TC	−0.270	0.088	9.525	0.002	0.76 (0.64–0.90)
TG	0.471	0.228	10.542	0.001	2.09 (1.34–2.28)
HDL-C	0.503	0.223	5.072	0.024	1.65 (1.06–2.56)
LDL-C	0.472	0.108	19.040	0.000	1.60 (1.29–1.98)
VLDL-C	0.035	1.163	0.001	0.976	1.03 (0.10–8.63)
WBC	0.048	0.020	5.626	<0.001	1.04 (0.95–1.09)
Recessive model					
Intercept	−1.697	0.327	26.921	<0.001	
GA + GG	0^b^				
AA	0.096	0.128	0.559	0.455	1.10 (0.85–1.41)
TC	−0.266	0.088	9,239	0.002	0.76 (0.64–0.91)
TG	0.745	0.230	10.516	0.001	2.10 (1.34–3.30)
HDL-C	0.499	0.223	5.010	0.025	1.64 (1.06–2.55)
LDL-C	0.467	0.108	18.734	<0.001	1.59 (1.29–1.97)
WBC	0.047	0.020	5.402	0.002	0.76 (0.64–0.91)

OR, 95%CI, and *p* were calculated by logistic regression analysis after adjustment for age and sex.

^b^With this category as the reference level, the parameter estimate is fixed at 0.

### 3.5 rs3733570 Polymorphism is associated with DKD in the presence of Hyperlipidemia

Given the distinct clinical features between T2DM and DKD, we further investigated the combined effect of the *QDPR* rs3733570 polymorphism and dyslipidemia on susceptibility to diabetic kidney disease (Table 5). Among participants with hyperlipidemia, carriers of the GA + GG genotypes demonstrated a significantly higher risk of developing DKD compared to those with T2DM alone (OR = 1.75; 95% CI: 1.11–2.76; *p* < 0.05). In addition, carriers of the AA genotype showed a significantly higher prevalence of DKD than the NC group under the Codominant model (OR = 1.80, 95% CI: 1.03–3.16, *p* < 0.05). Among individuals without hyperlipidemia, individuals carrying AA + GA genotypes had a higher prevalence of T2DM than the NC group, also reaching statistical significance under the dominant model (OR = 1.31; 95% CI: 1.01–1.72). The combined effect of the *QDPR* rs3733570 polymorphism and hypertension on susceptibility to diabetic kidney disease was further investigated; However, no statistically significant association was identified. Therefore, these results are provided in Supplementary Table 6.

**Table 5:** Distribution of genotypes in three groups of people with hyper lipidaemia.

Genotype	NC [*n* (%)]	T2DM [*n* (%)]	DKD [*n* (%)]	*p^a^*	OR (95%CI)	*p^b^*	OR (95%CI)	*p^c^*	OR (95%CI)
HLP (+)	155	327	179						
Codominant model									
GG	45 (30)	82 (24.9)	46 (25.4)						
GA	71 (45.2)	155 (47.1)	102 (56.3)	0.441	0.83 (0.52–1.32)	0.191	0.71 (0.42–1.18)	0.476	0.85 (0.55–1.32)
AA	39 (24.8)	90 (27.3)	31 (17.1)	0.817	0.94 (0.59–1.51)	0.037	1.80 (1.03–3.16)	0.078	1.62 (0.94–2.80)
Recessive model									
GA + GG	116 (75.2)	245 (72)	148 (81.7)						
AA	39 (24.8)	90 (27.3)	31 (17.1)	0.690	0.91 (0.59–1.41)	0.079	1.60 (0.94–2.72)	0.015	1.75 (1.11–2.76)
Dominant model									
GG	45 (30)	82 (24.9)	46 (25.4)						
AA+GA	110 (70)	245 (74.4)	133 (73.4)	0.357	1.22 (0.79–1.87)	0.495	1.18 (0.73–1.91)	0.878	0.96 (0.63–1.47)
HLP (−)	535	491	156						
Codominant model									
GG	174 (32.5)	133 (27.0)	44 (28.2)						
GA	246 (45.9)	240 (48.7)	67 (42.9)	0.444	1.18 (0.76–1.83)	0.733	0.92 (0.60–1.42)	0.444	1.18 (0.76–1.83)
AA	115 (20.5)	118 (23.9)	45 (28.8)	0.161	0.73 (0.47–1.13)	0.069	0.66 (0.42–1.03)	0.161	0.73 (0.47–1.13)
Recessive model									
GG+GA	420 (78.4)	373 (76)	111 (71.1)						
AA	115 (20.5)	118 (23.9)	45 (28.8)	0.209	0.82 (0.61–1.11)	0.034	1.11 (0.99–1.24)	0.228	0.78 (0.52–1.16)
Dominant model									
GG	174 (32.5)	133 (27.0)	44 (28.2)						
AA+GA	361 (66.4)	358 (72.6)	112 (71.7)	0.046	1.31 (1.01–1.72)	0.275	1.24 (0.84–1.84)	0.785	0.94 (0.63–1.41)

*p*^a^: T2DM VS NC; *p*^b^: DKD VS NC; *p*^c^: DKD VS T2DM.

## 4 Discussion

DKD is a major microvascular complication of diabetes mellitus and a leading cause of progressive kidney failure, contributing substantially to morbidity and premature mortality. Among the various factors implicated in the development of T2DM and DKD, genetic predisposition is regarded as a key determinant of individual susceptibility. From a molecular genetic perspective, specific gene variants, either independently or in combination, may confer an increased risk of disease onset. Dyslipidemia is increasingly recognized as a mechanistic contributor to DKD rather than merely a metabolic accompaniment. By disrupting endothelial integrity, compromising microvascular perfusion, and amplifying oxidative and inflammatory stress within the renal microenvironment, lipid abnormalities may accelerate structural and functional kidney injury. Therefore, the interplay between genetic susceptibility and disordered lipid metabolism may be central to understanding inter-individual differences in DKD onset, progression, and clinical expression. In this study, we examined the potential role of *QDPR* gene polymorphisms in DKD development. By sequencing the *QDPR* gene and performing a case–control genetic association analysis, we identified SNP rs3733570 as a variant linked to susceptibility to T2DM and, in the context of hyperlipidemia, to DKD in a Chinese Han population. Importantly, although *QDPR* has been implicated in BH4 metabolism and oxidative stress, previous clinical or genetic studies have not examined whether any *QDPR* polymorphisms, including rs3733570, are associated with type 2 diabetes or its complications, particularly DKD. Thus, to the best of our knowledge, our study is the first to explore and report this specific association. This is in line with previous evidence that genetic polymorphisms can modulate DKD risk, as variants in genes such as *TGF-β1*, *NQO1*, and *SLC2A1* have been associated with DKD in different ethnic groups. Our work therefore extends this body of literature by adding *QDPR* to the list of candidate genes that may contribute to the genetic susceptibility to diabetic complications. The rs3733570 variant is a synonymous substitution (c.489T>C) located in exon 4 of the *QDPR* gene (24). Although synonymous variants have long been regarded as functionally neutral (25, 26), increasing evidence suggests that they can affect gene expression and protein function through various mechanisms (27–29).

Specifically, rs3733570 alters the serine codon from TCA to TCG. While both codons encode the same amino acid, their usage frequency differs substantially in humans—TCA is more common (12.2 per 1,000 codons), whereas TCG is relatively rare (4.4 per 1,000 codons). This shift to a low-frequency codon may reduce translation efficiency due to the limited abundance of the corresponding tRNA, potentially leading to ribosome stalling (30), decreased protein production, and co-translational misfolding (31). Although a general relationship between rare codons and reduced translational output is well established, the precise mechanistic consequences of rs3733570—whether through altered mRNA stability, impaired folding, or other pathways—remain to be elucidated (32). Notably, previous research has linked rs3733570 with anorexia nervosa, further suggesting that this variant may influence *QDPR* function.

Notably, this synonymous variant exhibits significant population-specific differences in distribution: its minor allele frequency reaches approximately 50% in certain Asian populations, while being considerably lower in European populations. Given the crucial role of the *QDPR* gene in the regeneration of BH4 (33), this population-specific allelic distribution pattern suggests that the variant may have potential functional implications.

Our analysis revealed a statistically significant association between the rs3733570 variant and increased susceptibility to T2DM. Specifically, patients carrying the AA genotype or AA+GA genotypes demonstrated a higher risk of developing diabetes, suggesting that this variant exerts a genotype-dependent effect on disease pathogenesis. These findings align with an emerging pathophysiological framework that proposes *QDPR* dysfunction as a key contributor to T2DM development, primarily through the disruption of BH4 homeostasis. BH4 insufficiency leads to the uncoupling of eNOS (34), resulting in the generation of ROS and subsequent oxidative stress. Within pancreatic β-cells, BH4 deficiency exacerbates oxidative damage, further promoting ROS accumulation (35). This oxidative burden triggers endoplasmic reticulum (ER) stress, disrupting proteostasis through abnormal protein folding and activation of the unfolded protein response (UPR). Additionally, oxidative stress activates mitochondrial-mediated apoptotic pathways, contributing to β-cell dysfunction and death. Despite being triggered by distinct molecular perturbations—protein homeostasis disruption and mitochondrial dysfunction—these pathways converge at the level of downstream effectors to synergistically accelerate β-cell decompensation.

As a critical cofactor for TPH, the enzyme responsible for serotonin (5-HT) biosynthesis, BH4 regulates 5-HT production (36), which plays a pivotal role in modulating insulin secretion from pancreatic β-cells (37, 38). Based on this mechanistic framework, we propose that dysfunction in the *QDPR*-BH4 pathway contributes to systemic glucose dysregulation through three interconnected pathogenic cascades (39): (a) amplification of insulin resistance via proinflammatory signaling networks (40); (b) induction of β-cell failure through oxidative damage and impairment of cellular viability (41); and (c) attenuation of insulin secretion due to disruption of 5-HT-mediated paracrine and autocrine regulation.

These observations suggest potential therapeutic targets for T2DM management, particularly in addressing *QDPR* dysfunction and BH4 bioavailability. However, the clinical translatability of these findings requires rigorous validation through large-scale observational studies and interventional trials.

Furthermore, we observed a statistically significant association between rs3733570 and DKD in individuals with hyperlipidemia. Hyperlipidemia is a well-established risk factor for DKD (42) and can accelerate disease progression. This effect is likely mediated through the induction of oxidative stress and pro-inflammatory responses. Hypertriglyceridemia increases blood viscosity and promotes erythrocyte aggregation in capillaries, leading to impaired microvascular perfusion (43). Concurrently, hypercholesterolemia exacerbates ischemia–reperfusion injury and stimulates inflammatory responses within the microvasculature, collectively accelerating DKD onset and progression (44). Clinical studies have demonstrated that lipid-lowering agents, such as statins, can provide renal protection by significantly reducing proteinuria in patients with DKD (45–47). As previously discussed, *QDPR* plays a crucial role in maintaining BH4 levels, which directly suppress ROS generation. BH4 deficiency leads to uncoupling of nitric oxide synthase (NOS) (48), further increasing ROS production. In addition to its antioxidant properties, BH4 serves as an essential cofactor for tryptophan hydroxylase (TPH) (49), the rate-limiting enzyme in serotonin (5-HT) biosynthesis (50). Emerging evidence indicates that 5-HT contributes to DKD progression by promoting excessive type IV collagen production in glomerular mesangial cells, thereby accelerating glomerulosclerosis (51). It also regulates *TGF-β* expression, a key mediator of fibrosis, and influences macrophage secretion of proinflammatory cytokines (52), amplifying renal inflammation. These findings provide novel theoretical insights into the pathogenesis of DKD. Finally, although we identified rs3733570 as a potential risk factor, the underlying molecular mechanisms by which this synonymous variant might affect *QDPR* function (and thereby influence disease risk) remain unclear. Further functional experiments (*in vitro* and *in vivo*) are needed to elucidate how rs3733570 (or linked variants) might alter *QDPR* expression, enzyme activity, or BH4 metabolism.

## 5 Limitations

While the present study provides valuable insights into the association between *QDPR* rs3733570 and diabetic conditions, several limitations should be acknowledged. First, the study population was restricted to Chinese Han individuals. Although this relative homogeneity may help reduce background genetic variability, it may also limit the generalizability of our findings to other ethnic groups. Second, the cross-sectional design of our study precludes conclusions about causality or temporality. It cannot be determined whether the *QDPR* variant contributes to the onset of T2DM/DKD or if it influences the progression of DKD over time. Prospective longitudinal studies are needed to address this. Third, although several biochemical parameters were evaluated, environmental and lifestyle factors—such as dietary patterns, physical activity, body mass index (BMI), and insulin resistance indices (e.g., HOMA-IR)—were not comprehensively assessed. These unmeasured variables may contribute to variability in DKD risk. Future studies should therefore incorporate a broader range of clinical and lifestyle covariates to better control for potential confounding factors. Finally, only one SNP within the *QDPR* gene was selected for further analysis. As a result, a meaningful linkage disequilibrium (LD) analysis could not be performed in the present study. Future investigations including multiple SNPs across the QDPR locus are needed to provide a more comprehensive assessment of its genetic contribution to DKD susceptibility.

## 6 Conclusion

The study identified, for the first time, the genetic variant in the *QDPR* gene represent novel susceptibility markers for Type 2 diabetic mellitus and diabetic kidney disease in a Chinese Han population, with a more pronounced effect observed in individuals with hyperlipidemia. Although rs3733570 is a synonymous variant, it may contribute to disease susceptibility by affecting BH4 availability, oxidative stress, and related metabolic pathways, thereby implicating the *QDPR*/BH4 axis in the pathogenesis of DKD. Because this is the first study to look into the role of the QDPR gene polymorphisms in diabetic mellitus, there are no comparable studies to compare our findings to. Further studies in larger and more diverse populations are required to validate these associations and facilitate a detailed understanding of the precise biological mechanisms, thereby informing potential therapeutic strategies, such as whether improving endothelial function or antioxidant capacity could mitigate the risk conferred by this variant.

## Data Availability

The raw data supporting the conclusion of this article will be made available by the authors, without undue reservation.
