# Supplementary material for: QDPR rs3733570 polymorphism is associated with type 2 diabetes and diabetic kidney disease accompanied by hyperlipidemia in the Chinese Han population
Source: Front Med (Lausanne). 2026 May 12;13:1811028. doi: 10.3389/fmed.2026.1811028 (PMC13201145; doi:10.3389/fmed.2026.1811028)
Supplement: Supplementary file 1 [file Table_1.docx]

Supplementary Material

| Supplementary Table 1 Analysis for Screening Potential Risk Factors of T2DM | | | | | |
| --- | --- | --- | --- | --- | --- |
| Variable | β | S.E | *Wald*$x^{2}$ | *P* | OR(95% *CI*) |
| Sex (Male) | 0.181 | 0.345 | *3.890* | 0.049 | 1.19(0.60～2.35) |
| Coronary heart disease | -0.691 | 0.349 | *3.929* | 0.047 | 0.50(0.25～0.99) |
| Cerebrovascular diseases | -0.274 | 0.347 | *0.621* | 0.431 | 0.76(0.38～1.50) |
| Hyperlipidemia | 0.615 | 0.382 | *2.596* | 0.107 | 1.85(0.87～3.90) |
| Hypertension | -0.256 | 0.335 | *0.584* | 0.445 | 0.77(0.40～1.49) |
| FSB | -0.104 | 0.045 | 5.253 | 0.022 | 0.90(0.82～0.98) |
| TC | 0.388 | 0.273 | 2.011 | 0.156 | 1.47(0.86～2.51) |
| TG | -0.443 | 0.526 | 0.708 | 0.400 | 0.64(0.22～1.80) |
| HDL-C | -1.147 | 0.625 | 3.366 | 0.067 | 0.31(0.93～1.08) |
| LDL-C | -0.601 | 0.319 | 3.545 | 0.060 | 0.54(0.29～1.02) |
| VLDL-C | 0.229 | 2.829 | 0.007 | 0.935 | 1.25(0.52～3.12) |
| WBC | -0.037 | 0.051 | 0.527 | 0.468 | 0.96(0.87～1.06) |
| RBC | -0.910 | 0.462 | 1.793 | 0.181 | 0.53(0.21～1.33) |
| Hb | 0.039 | 0.015 | 0.800 | 0.371 | 1.01(0.98～1.04) |
| P<0.05 is considered to be significant | | | | | |

| Supplementary Table 2 Analysis for Screening Potential Risk Factors of DKD | | | | | |
| --- | --- | --- | --- | --- | --- |
| Variable | β | S.E | Wald$x^{2}$ | *P* | OR(95% CI) |
| Onset of diabetic | 0.048 | 0.129 | 25.047 | ＜0.001 | 1.04(1.03～1.06) |
| T2DM duration | -0.077 | 0.113 | 34.099 | ＜0.001 | 0.92(0.90～0.95) |
| Coronary heart disease | -0.323 | 0.169 | 3.634 | 0.057 | 0.72 (0.52～1.01) |
| Cerebrovascular diseases | 0.662 | 0.165 | 16.109 | ＜0.001 | 1.93(1.04～2.68) |
| Hyperlipidemia | 0.375 | 0.186 | 4.082 | 0.043 | 1.45(1.01～2.09) |
| Hypertension | -1.080 | 0.195 | 30.829 | 0.000 | 0.34(0.23～0.49) |
| FSB | -0.856 | 0.043 | 36.047 | ＜0.001 | 2.23(2.05～2.43) |
| TC | -0.278 | 0.093 | 9.041 | 0.003 | 0.75(0.63～0.90) |
| TG | -0.006 | 0.108 | 0.003 | 0.954 | 0.99(0.80～1.22) |
| HDL-C | -0.333 | 0.304 | 1.200 | 0.273 | 0.71(0.39～1.30) |
| LDL-C | 0.143 | 0.136 | 1.100 | 0.294 | 1.15(0.88～1.50) |
| VLDL-C | 0.183 | 0.690 | 0.071 | 0.790 | 1.20(0.31～4.64) |
| WBC | -0.064 | 0.029 | 4.947 | 0.026 | 0.93(0.88～0.99) |
| RBC | 0.356 | 0.276 | 1.670 | 0.956 | 1.42(0.83-2.42) |
| Hb | 0.015 | 0.008 | 3.176 | 0.075 | 1.01(0.99-1.03) |
| P<0.05 is considered to be significant | | | | | |

| Supplementary Table 3 Unconditional multifactorial logistic regression analysis of risk factors for diabetic kidney disease | | | | | |
| --- | --- | --- | --- | --- | --- |
| Variables | β | S.E | Wald$x^{2}$ | *P* | OR(95%CI) |
| Codominant model |  |  |  |  |  |
| Intercept | 1.306 | 0.633 | 4.249 | 0.039 |  |
| GG | 0^b^ |  |  |  |  |
| GA | 0.134 | 0.181 | 0.055 | 0.458 | 1.14(0.80～1.62) |
| AA | 0.234 | 0.211 | 1.226 | 0.268 | 1.26(0.83～1.91) |
| TC | -0.334 | 0.091 | 13.579 | ＜0.001 | 0.71(0.59～0.89) |
| TG | 0.072 | 0.105 | 0.465 | 0.495 | 1.07(0.87～1.32) |
| HDL-C | -0.034 | 0.293 | 0.013 | 0.909 | 0.96(0.54～1.71) |
| LDL-C | 0.257 | 0.130 | 3.916 | 0.048 | 1.29(1.01～1.66) |
| VLDL-C | 0.026 | 0.669 | 0.001 | 0.970 | 1.02(0.27~3.80) |
| Coronary heart disease | -0.221 | 0.161 | 1.896 | 0.168 | 0.80(0.58～1.09) |
| Hyperlipidemia | 0.356 | 0.177 | 4.065 | 0.044 | 1.42(1.01～2.01) |
| WBC | -0.050 | 0.026 | 3.670 | 0.055 | 0.95(0.90~1.01） |
| Onset of diabetes | 0.130 | 0.011 | 133.266 | ＜0.001 | 0.90(0.88~0.92) |
| OR,95%CI,and *P* were calculated by logistic regression analysis after adjustment for age and sex | | | | | |

| Supplementary Table 4 Unconditional multifactorial logistic regression analysis of risk factors for diabetic kidney disease | | | | | |
| --- | --- | --- | --- | --- | --- |
| Variables | β | S.E | Wald$x^{2}$ | *P* | OR(95%CI) |
| Recessive model |  |  |  |  |  |
| Intercept | 1.306 | 0.633 | 4.249 | 0.039 |  |
| GA+GG | 0^b^ |  |  |  |  |
| AA | -0.514 | 0.182 | 0.714 | 0.398 | 0.85(0.61～1.12) |
| TC | -0.307 | 0.093 | 10.797 | 0.001 | 0.73(0.61～0.88) |
| TG | 0.020 | 0.107 | 0.036 | 0.851 | 1.02(0.82～1.25) |
| HDL-C | -0.273 | 0.298 | 0.839 | 0.360 | 0.76(0.42～1.36) |
| LDL-C | 0.257 | 0.130 | 3.916 | 0.048 | 1.29(1.01～1.66) |
| VLDL-C | -0.010 | 0.681 | 0.001 | 0.988 | 0.99(0.26~3.76) |
| Coronary heart disease | 0.304 | 0.166 | 3.345 | 0.067 | 0.80(0.58～1.09) |
| Hyperlipidemia | -0.350 | 0.183 | 3.680 | 0.055 | 0.74(0.49～1.01) |
| WBC | -0.062 | 0.029 | 4.568 | 0.033 | 0.94(0.88~0.99） |
| Onset of diabetes | 0.126 | 0.012 | 119.780 | ＜0.001 | 1.13(1.10~1.16) |
| OR,95%CI,and *P* were calculated by logistic regression analysis after adjustment for age and sex | | | | | |

| Supplementary Table 5 Unconditional multifactorial logistic regression analysis of risk factors for diabetic kidney disease | | | | | |
| --- | --- | --- | --- | --- | --- |
| Variables | β | S.E | Wald$x^{2}$ | *P* | OR(95%CI) |
| Dominant model |  |  |  |  |  |
| Intercept | 1.306 | 0.633 | 4.249 | 0.039 |  |
| GG | 0^b^ |  |  |  |  |
| AA+GA | 0.228 | 0.174 | 1.718 | 0.001 | 0.73(0.60～0.88) |
| TC | -0.311 | 0.094 | 10.977 | 0.001 | 1.03(0.83～1.27) |
| TG | 0.031 | 0.107 | 0.082 | 0.774 | 1.03(0.83～1.27) |
| HDL-C | -0.283 | 0.299 | 0.901 | 0.342 | 0.75(0.42～1.35) |
| LDL-C | 0.180 | 0.134 | 1.810 | 0.179 | 1.19(0.92～1.55) |
| VLDL-C | -0.065 | 0.681 | 0.009 | 0.924 | 0.93(0.24~3.56) |
| Coronary heart disease | 0.306 | 0.166 | 3.402 | 0.065 | 1.35(0.98～1.88) |
| Hyperlipidemia | -0.361 | 0.183 | 3.903 | 0.048 | 0.69(0.48～0.99) |
| WBC | -0.059 | 0.029 | 4.293 | 0.038 | 0.94(0.89~0.99） |
| Onset of diabetes | 0.126 | 0.013 | 44.077 | 0.000 | 0.91(0.89~0.94) |
| OR,95%CI,and *P* were calculated by logistic regression analysis after adjustment for age and sex | | | | | |

| Supplementary Table 6 Distribution of genotypes in three groups of people with Hypertension | | | | | | | | | | | | | | | | | |  |  |
| --- | --- | --- | --- | --- | --- | --- | --- | --- | --- | --- | --- | --- | --- | --- | --- | --- | --- | --- | --- |
| GENOTYPY | | NC (%) | T2DM (%) | | DKD (%) | | *P^a^* | | OR (95%CI) | | *P^b^* | | OR (95%CI) | | *P^c^* | | OR (95%CI) | |  |
| HPN(+) | | 405 | 538 | | 277 | |  | |  | |  | |  | |  | |  | |  |
| Codominant model | |  | |  | |  | |  | |  | |  | |  | |  | |  | |
| GG | | 122(30.1) | 80(25.0) | | 74(26.9) | |  | |  | |  | |  | |  | |  | |  |
| GA | | 188(46.4) | 122(50.7) | | 145(52.3) | | 0.248 | | 1.23(0.86-1.77) | | 0.977 | | 1.00(0.65-1.55) | | 0.859 | | 0.96(0.68-1.37) | |  |
| AA | | 95(23.5) | 130(24.3) | | 58(20.8) | | 0.083 | | 1.31(0.96-1.78) | | 0.192 | | 1.272(0.88-1.82) | | 0.336 | | 0.814(0.53-1.37) | |  |
| Recessive model | |  |  | |  | |  | |  | |  | |  | |  | |  | |  |
| GA+GG | | 310(69.5) | 408(75.7) | | 219(67.2) | |  | |  | |  | |  | |  | |  | |  |
| AA | | 95(23.5) | 130(24.3) | | 58(20.8) | | 0.801 | | 1.04(0.76-1.40) | | 0.439 | | 0.864(0.97-1.25) | | 0.301 | | 0.831(0.58-1.18) | |  |
| Dominant model | |  |  | |  | |  | |  | |  | |  | |  | |  | |  |
| GG | | 122(30.1) | 80(25.0) | | 74(26.9) | |  | |  | |  | |  | |  | |  | |  |
| AA+GA | | 283(69.9) | 252(75.0) | | 133(73.1) | | 0.086 | | 0.77(0.58-1.03) | | 0.334 | | 0.846(0.60-1.18) | | 0.616 | | 1.08(0.78-1.51) | |  |
| HPN (-) | | 285 | 280 | | 59 | |  | |  | |  | |  | |  | |  | |  |
| Codominant model | |  |  | |  | |  | |  | |  | |  | |  | |  | |  |
| GG | | 97(34.0) | 80(28.5) | | 15(25.4) | |  | |  | |  | |  | |  | |  | |  |
| GA | | 129(45.3) | 122(43.5) | | 24(40.6) | | 0.441 | | 0.83(0.52-1.32) | | 0.191 | | 1.40(0.84-2.34) | | 0.511 | | 0.86(0.55-1.33) | |  |
| AA | | 59(20.7) | 78(23.0) | | 20(34.0) | | 0.376 | | 1.19(0.75-1.89) | | 0.550 | | 0.82(0.44-1.53) | | 0.505 | | 1.18(0.72-1.91) | |  |
| Recessive model | |  |  | |  | |  | |  | |  | |  | |  | |  | |  |
| GA+GG | | 226(79.3) | 202(77) | | 39(66.0) | |  | |  | |  | |  | |  | |  | |  |
| AA | | 59(20.7) | 78(23.0) | | 20(34.0) | | 0.209 | | 1.32(0.61-1.61) | | 0.034 | | 1.11(0.99-1.24) | | 0.228 | | 0.78(0.52-1.16) | |  |
| Dominant model | |  |  | |  | |  | |  | |  | |  | |  | |  | |  |
| GG | | 97(34.0) | 80(28.5) | | 15(25.4) | |  | |  | |  | |  | |  | |  | |  |
| AA+GA | | 188(66) | 200(71.5) | | 44(74.6) | | 0.021 | | 0.83(0.52-1.32) | | 0.191 | | 1.40(0.84-2.34) | | 0.476 | | 1.17(0.75-1.82) | |  |
| *P^a^*:T2DM VS NC *P^b^* :DKD VS NC,*P^c^* : DKD VS T2DM. | | | | | | | | | | | | | | | | | |  |  |
